# Clinical evaluation of regenerative potential of type I collagen membrane along with xenogenic bone graft in the treatment of periodontal intrabony defects assessed with surgical re-entry and radiographic linear and densitometric analysis

**DOI:** 10.4103/0972-124X.65432

**Published:** 2010

**Authors:** N. K. Sowmya, A. B. Tarun Kumar, D. S. Mehta

**Affiliations:** Department of Periodontology and Implantology, Bapuji Dental College and Hospital, Davanagere - 577 004, India

**Keywords:** Bone grafts, collagen membrane, guided tissue regeneration, periodontal regeneration

## Abstract

**Background and Objectives:**

The primary goal of periodontal therapy is to restore the tooth supporting tissues lost due to periodontal disease. The aim of the present study was to compare the efficacy of combination of type I collagen (GTR membrane) and xenogenic bone graft with open flap debridement (OFD) in treatment of periodontal intrabony defects.

**Materials and Methods:**

Twenty paired intrabony defects were surgically treated using split mouth design. The defects were randomly assigned to treatment with OFD + collagen membrane + bone graft (Test) or OFD alone (Control). The clinical efficacy of two treatment modalities was evaluated at 9 month postoperatively by clinical, radiographical, and intrasurgical (re-entry) parameters. The measurements included probing pocket depth (PD), clinical attachment level (CAL), gingival recession (GR), bone fill (BF), bone density (BD) and intra bony component (INTRA).

**Results:**

The mean reduction in PD at 0–9 month was 3.3±0.82 mm and CAL gain of 3.40±1.51 mm occurred in the collagen membrane + bone graft (Test) group; corresponding values for OFD (Control) were 2.20±0.63 mm and 1.90±0.57 mm. Similar pattern of improvement was observed when radiographical and intra-surgical (re-entry) post operative evaluation was made. All improvement in different parameters was statistically significant (*P*< 0.01).

**Interpretation and Conclusion:**

Treatment with a combination of collagen membrane and bone graft led to a significantly more favorable clinical outcome in intrabony defects as compared to OFD alone.

## INTRODUCTION

Periodontal disease comprises a group of different disorders, most of which affect the supporting structures of the teeth and may ultimately result in the loss of teeth. During destructive periodontal disease, the connective tissue attachment of the tooth is destroyed leading to pocket formation and concomitantly, alveolar bone resorption.

Complete regeneration of the functional attachment apparatus has remained an elusive goal of the periodontal therapy, and currently the major progress is being made to achieve this end by utilising various regenerative procedures such as bone grafting, GTR techniques and combination therapy.[1,2] Both animal and human studies have shown that the combination of GTR procedure and bone grafting greatly enhances the regenerative outcome.

The use of type I collagen for GTR has an added advantage because, it is uniquely involved in the binding of cells, particularly fibroblasts and osteoblasts, thus promoting periodontal regeneration.[3,4]

Xenografts used in the treatment of infrabony defects can be both bovine bone and natural coral, these are also referred to as an anorganic bone, since proprietary processes are suggested to remove all cells and proteinaceous material, leaving behind an inert absorbable bone scaffolding upon which revascularization, osteoblast migration, and woven bone formation supposedly occur.

The purpose of this study was to compare and evaluate regenerative potential of type I collagen resorbable membrane (Healiguide^®^) with Xenogenic bone graft (Osseograft®) with access flap alone in treatment of periodontal intrabony defects assessed with surgical re-entry procedure and densitometric analysis using Image J analysis software.

## MATERIALS AND METHODS

Total of ten patients (three males and seven females) with mean age group of 45.6±2.8 years were selected from the Out Patient Department of Periodontics, Bapuji Dental College and Hospital, Davangere. The selection criteria include patients with good systemic health with no contraindication for periodontal surgery, non smokers and clinically having bilateral infrabony pockets of more than 5mm depth, with radiographic evidence of vertical bone loss. Verbal and written informed consent was obtained from all patients before the commencement of the study. Split mouth design was planned and the sites were divided randomly as either test or control.[5]

### Clinical parameters

Clinical examination was performed at baseline and nine months after the surgical procedure. The oral hygiene status was evaluated by the Plaque index[6] (PI) as an expression of the level of an individual’s supragingival plaque accumulation. Gingival inflammation was assessed by the Gingival index[7] (GI). Clinical outcome variables like Probing Depth[8] (PD), Clinical attachment level[9] (CAL) and Gingival recession[10] (GR) were measured using the acrylic occlusal stent.

### Radiographic parameters

Intra-oral periapical (IOPA) radiographs of the selected sites were taken using long cone paralleling (LCP) technique with 70 KVp, 10 mA and exposure time of 0.8 seconds and were subjected to Linear measurement and Densitometric analysis at baseline and 9 months post surgery, using the Image J Analysis software and postoperative defect fill was calculated.

### Materials used in the study

*Osseograft*^®^: Osseograft^®^ demineralized bone matrix (DMBM) is a bio-resorbable xenograft composed of type -I collagen which is used for regeneration of osseous defects in periodontal as well as oral and maxillofacial surgeries.

*Healiguide*^®^: Is a bio-resorbable type-I collagen membrane that is indicated for guided tissue regeneration in periodontal osseous defects to enhance regeneration of lost bone. It is available in the form of a thin sheet enclosed in a box and available as three sizes (15 × 20 mm^2^, 20 × 30 mm^2^, and 30 × 40 mm^2^).

### Presurgical procedure

Following initial examination and treatment planning, the selected patients underwent Phase I therapy. Detailed instructions regarding self-performed plaque control measures were given. After four weeks, only those patients maintaining optimum oral hygiene were subjected to the surgical procedure.

### Surgical procedure

A similar surgical protocol was followed for all the cases. Periodontal surgical procedures were performed on an outpatient basis under aseptic conditions. After providing local anesthesia to the subjects, crevicular incisions were made, and full-thickness mucoperiosteal flaps were elevated. Meticulous defect debridement and root planning were carried out to remove visible subgingival plaque, calculus, inflammatory granulation tissue, and pocket epithelium. The surgical sites were rinsed thoroughly with sterile saline, and care was taken to keep the area free of saliva and blood.

Baseline Intrasurgical Parameters were recorded i.e., from CEJ to base of the defect (CEJ to BD) and CEJ to alveolar bone crest (CEJ to BC) by using Williams graduated periodontal probe and the intrabony component INTRA was defined as distance from (CEJ to BD) - (CEJ to BC). These parameters were required to compare the regenerative potential of the materials during surgical re-entry.

### Preparation and application of Osseograft^®^

The required quantity of the bone graft material was transferred from the vial to the dappen dish and mixed with normal saline. When it became a cohesive mass, it was delivered in small increments into the defect, taking care not to overfill it.

### Preparation of the Healiguide^®^ GTR membrane

Template was prepared according to the morphology of defect and subsequently the membranes were trimmed according to the template. It was extended approximately 2 mm beyond the bony margin and was hydrated in normal saline for few seconds. Later hydrated membrane was adapted over the defect which was filled with Osseograft^®^.

**Figure 1:**
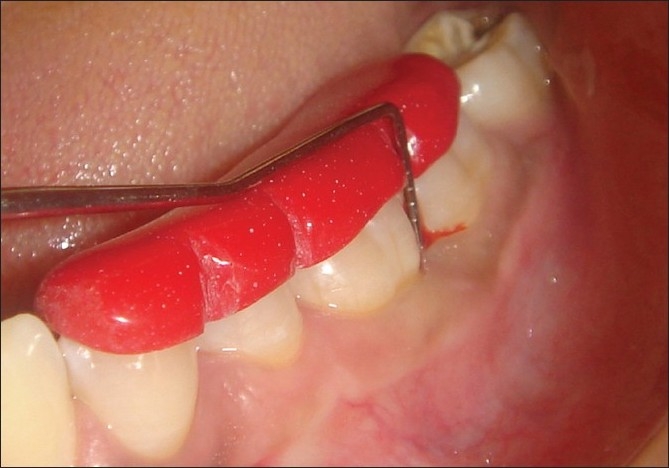
Measuring the probing pocket depth using Williams graduated probe

**Figure 2:**
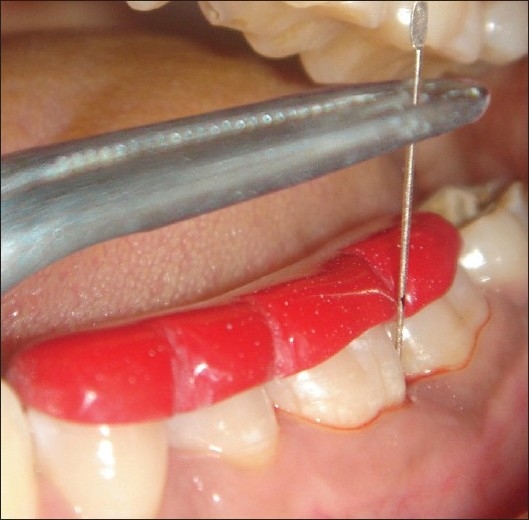
Measuring the CAL using silver point and occlusal stent

**Figure 3:**
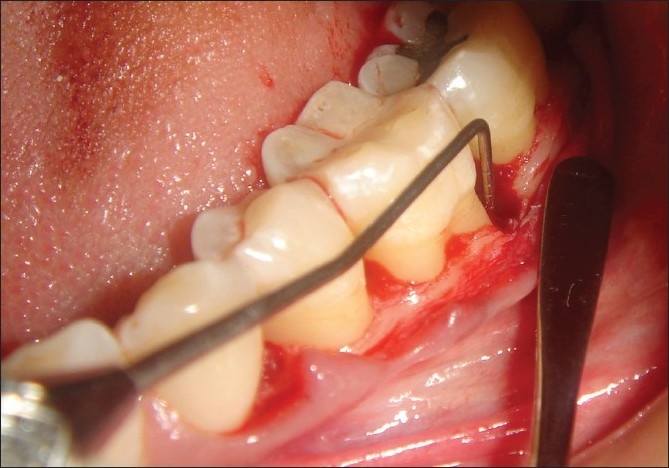
Measuring the intra-surgical defect depth

**Figure 4:**
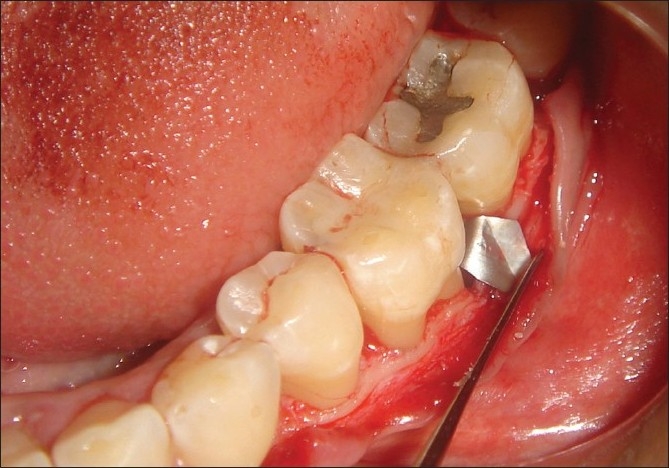
Template adapted

**Figure 5:**
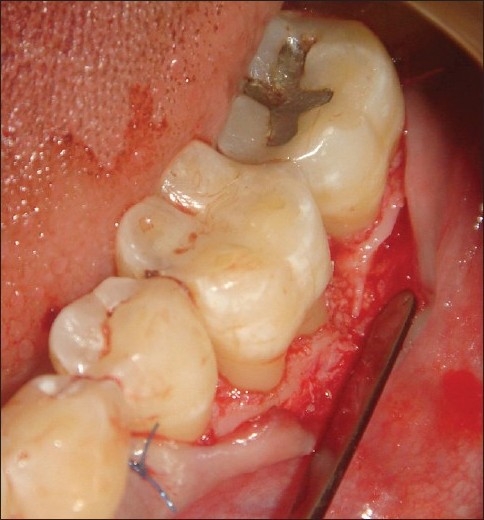
Bone graft placed

**Figure 6:**
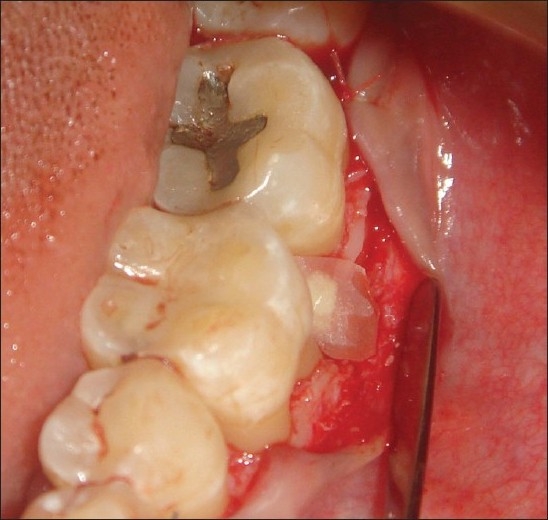
Membrane adapted

**Figure 7:**
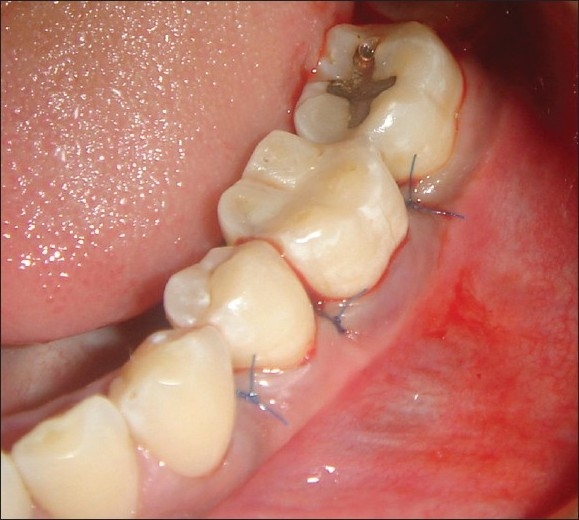
Sutures placed

The mucoperiosteal flaps were repositioned and secured in place by interrupted suture using the black braided (4-0) silk [Figures 1–7]. The surgical area was protected and covered with non-eugenol dressing (Coe-Pack®, G C America Inc, USA). All patients were prescribed systemic Doxycycline HC1 (Doxy-T 100 mg) 200 mg for first day, followed by 100 mg/day for six days and Diclofenac sodium (Diclogesic) 50 mg thrice daily for three days.

### Post surgical procedure

After one week following surgery, the periodontal dressing and sutures were removed and the area was irrigated thoroughly with saline. Patients were evaluated clinically and radiographically at nine months, postoperatively. At this visit, oral hygiene instructions were reinforced and scaling was done if necessary.

### Surgical re-entry procedure

**Figure 8:**
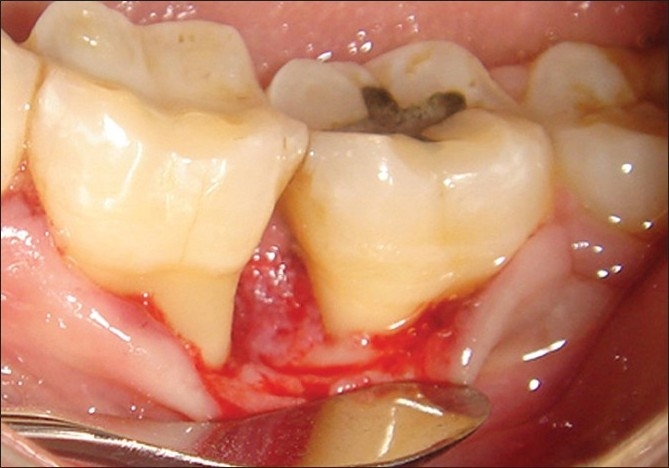
Surgical re-entry

After nine months postoperatively, the treated sites were anesthetized, crevicular incisions were given on the facial and lingual/palatal sides reaching the tip of the interdental papilla. Full thickness mucoperiosteal flaps were reflected using the periosteal elevator, loose soft tissue was removed and intra -surgical re-entry measurements were recorded.After irrigating the site with saline, the flaps were sutured back with black braided (4-0) silk suture and covered with a non-eugenol periodontal dressing (Coe-Pack®, G C America Inc, USA) [Figure 8].

One week following surgery, the periodontal dressing and the sutures were removed and the area was thoroughly irrigated with saline and oral hygiene instructions were reinforced.

### Interpretation of radiographs

**Figure 9:**
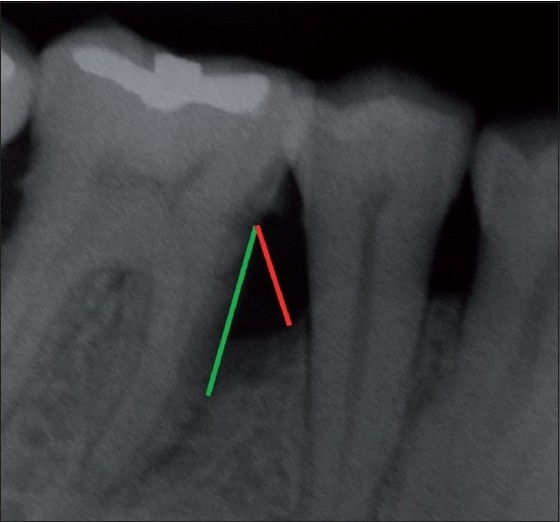
Linear radiographic interpretation with Computer Image analysis software. (Green line indicates CEJ to base of defect, red line indicates CEJ to alveolar bone crest)

Standardized intra oral periapical (IOPA) radiographs were taken at baseline and 9 months post-operatively for each defect. Interpretation of radiographs was carried out by means of Image J analysis for both linear and density measurements.[11] [Figures 9 and 10a and 10b]

**Figure 10a:**
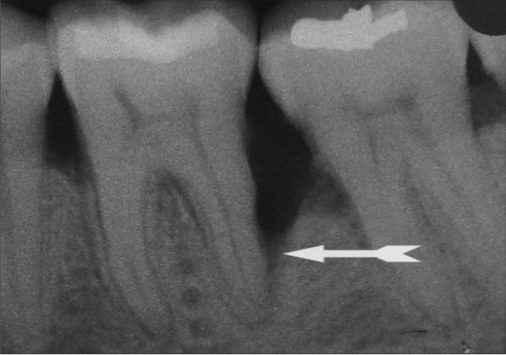
Preoperative radiographs

**Figure 10b:**
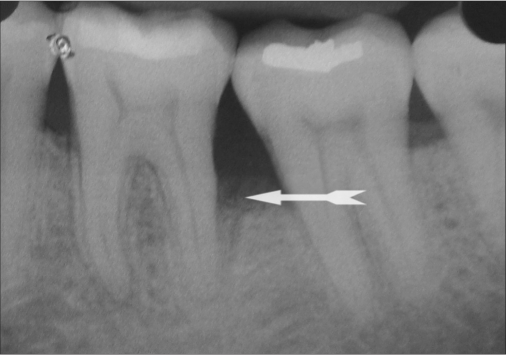
Postoperative radiographs

### Statistical analysis

Descriptive data that included mean ± SD and percentages were calculated for each clinical and radiographic parameter, at baseline and at different time intervals. Nonparametric tests were used for Intra (Wilcoxon’s signed rank test) and Inter (Mann-Whitney U test) group comparisons. A level of significance was set at the probability value of *P*<0.05.

## RESULTS

All ten patients completed the nine-month study period. Both test and control group sites in all ten patients healed uneventfully. No evidence of flap dehiscence or infection was reported in any of the surgical site. The soft tissue response, in both tests and control groups, was excellent.

Both test and control groups showed significant pocket depth reduction at nine months when compared to baseline [Table 1]. The mean pocket depth reduction in test group was 3.30±0.82 mm and in control site was 2.20±0.63 mm. The difference between the groups was statistically significant (*P*<0.01) in favor of test groups [Table 1].

**Table 1:** Clinical parameter at baseline and 9 month of both groups

Parameters	BL	9 month	BL-9 month	Significance Test vs. Control
Probing depth				
Test	7.40±1.41	3.60±1.01	3.30±0.82	<0.01S
Control	8.20±6.2	6.00±1.56	2.20±0.63	
CAL				
Test	12.40±1.26	9.00±2.00	3.40±1.51	<0.01S
Control	11.50±1.78	9.60±1.78	1.90±0.57	
Gingival recession				
Test	5.10±0.99	5.90±1.73	‒0.80±1.14	NS
Control	3.30±2.21	4.00±2.40	‒0.70±0.82	

BL - Baseline, CAL - Clinical attachment level, S - Significant, NS - Nonsignificant

The gain in CAL was 3.40±1.51 mm for test, and for control group it was 1.90±0.57 mm; the difference was statistically significant (*P*<0.01) in favor of test groups [Table 1]. However, the postoperative gingival recession (GR) in both test and control groups was comparable i.e., ‒0.80±1.14 mm and ‒0.70±0.82 mm, respectively, without any significant difference between the two groups [Table 1].

Radiographically, the bone fill (BF) in test and control groups was recorded as 1.94±0.59 mm and 0.38±0.46 mm, respectively, after nine months, postoperatively [Table 2]; the difference between the two groups was statistically significant (*P*<0.01) in favor of test group. Similarly when both groups were compared with respect to the density of the regenerated bone at the defect site, it was 5.22±0.86 mm^2^ in test group as compared to the control (0.65±0.36 mm^2^); the difference being statistically significant (*P*<0.01) in favor of test group [Table 3].

**Table 2:** Radiographic measurements at baseline and 9 month of both groups

Parameters	BL	9 month	BL-9 month	Significance Test vs. Control
CEJ TOBD(A)				
Test	6.60±2.68	3.67±1.92	3.03±1.17	<0.01S
Control	6.85±2.38	6.11±1.96	0.74±0.56	
CEJ TOBC(B)				
Test	3.22±1.43	2.59±1.39	0.70±0.88	<0.01S
Control	3.01±1.12	2.65±0.99	0.36±0.24	
INTRA(A-B)				
Test	3.38±1.51	1.23±0.82	1.94±0.95	<0.01S
Control	3.84±2.25	3.46±1.87	0.38±0.46	

**Table 3:** Comparison of radiographic amount of mean change in density (in mm^2^)

Density	BL	9 month	BL-9 month	Significance Test vs. Control
Test	7.71±0.89	12.93±0.94	5.22±0.86	<0.01S
Control	8.26±0.89	8.91±0.75	0.65±0.36	

**Table 4:** Intrasurgical measurements at BL, 9 month of both groups

Parameters	BL	9 month	BL-9 month	Significance Test vs. Control
CEJ TOBD(A)				
Test	8.10±1.37	5.10±1.10,	3.00±0.822	<0.01S
Control	8.80±1.81	7.90±1.85	0.90±0.32	
CEJ TOBC(B)				
Test	4.80±1.14	4.00±1.15	0.80±0.63	NS
Control	4.00±0.82	3.70±0.82	0.30±0.48	
INTRA(A-B)				
Test	3.30±0.95	1.10±0.99	2.20±1.03	<0.01S
Control	4.80±1.48	4.20±1.69	0.60±0.52	

The intra surgical component of the regenerated tissue (INTRA) in test and control groups was recorded as 2.20±1.03 mm and 0.60±0.52 mm, respectively, on re-entry procedure carried out at nine months, postoperatively [Table 4]. The difference between the two groups was statistically significant in favor of test group.

## DISCUSSION

The ultimate goal of periodontal therapy is to provide a dentition that functions in health and comfort for the life of the patient. A shortcoming encountered with the currently available modalities of periodontal regeneration is the limited predictability. Even though various regenerative procedures like GTR, osseous grafting or the combination of both, have been shown to be effective in promoting clinical, radiographical, and histologic periodontal regeneration, complete restoration of the attachment apparatus in every treated defect is still not a reality. The present study was designed to compare the combined effect of GTR+ bone graft (test group) with the open flap debridement (OFD) in the treatment of periodontal intrabony defects.

The split mouth design was adopted in the present study because of the advantages of using subjects as their own control which helps control for inter-patient variability by treating paired defects in the same subject. Since there is no spillover effect, the treatment effect in one area of the mouth should not influence the effect of other treatment in the mouth.[5] Furthermore, only three wall intrabony defects were included in the present study because bone regeneration is believed to be improved with increasing number of bony walls facing the root surface. The three wall defect allows better containment and increased blood supply to the graft.[12]

The clinical outcome measures for determining the effect of therapy on the anatomical defects produced by periodontal diseases are probing pocket depth and CAL and both these treatment measures are considered as the widely accepted therapeutic end point after periodontal regenerative therapy.[13,14] In the present study, the combination of collagen membrane and xenogenic bone graft used in the treatment of periodontal intrabony defects in a nine-month study, demonstrated positive clinical outcome (for example, reduction in probing pocket depth and gain in CAL). The results obtained are comparable with those of previous studies by Camello *et al*.,[15] Paolantanio *et al*.,[16] and Camargo *et al*.[17]

Radiographic monitoring of alveolar bone changes following regenerative procedures is a noninvasive, painless alternative to direct bone measurements; regeneration in periodontal defects is usually measured by BF in angular defects.[18] In the present study, the radiographic assessment was carried out by analyzing linear distances and bone density (BD) changes using Image J analysis as the digitizing unit gives the precise value.[19,20] There was significant BF in the test group than in the control group when the bone level was compared radiographically from baseline to 9 months, postoperatively. Similar trend was observed when BD was compared between test and control sites. This observation was in agreement with the previous studies by Chen *et al*,[21] Wang *et al*.[22] Reentry procedures have the potential to determine if the new tissue has formed at the surgically operated site. It allows direct comparison of present bone level to the bone level at the time of regenerative surgery giving direct assessment of BF (INTRA).[18] The results in the present study showed substantial BF (INTRA) in the test group as compared to the OFD at the end of nine-month study period. The outcome obtained in our study is comparable with those of previous studies.[18,23–27]

The selection of regenerative material and technique in the present study was based on some evidence and clinical experience. The bone graft material (Osseograft^®^) acts as a filler material in defect, supporting the overlying GTR membrane and avoiding the membrane collapse. It acts as a bone substitute that promotes the formation of native bone as they function as bioabsorbable matrix for healing while encouraging new bone formation by osteoconductive/osteinductive bioactivity. It also acts as a framework into which bone forming cells and blood vessels integrate leading to the formation of healthy new bone and subsequent repair of the osseous defect.

The collagen (type I) was selected as a GTR membrane in the present study was based on the following facts:[4]

1. Type I collagen is the main constituent of periodontal connective tissue and therefore would seem to be an appropriate barrier in the GTR technique.
2. It is bio-absorbable so can act as a barrier analogous to a nonresorbable membrane and it is either incorporated into the healing connective tissues or is degraded by macrophages in 6–8 weeks.
3. Exogenous collagen is chemotactic for periodontal ligament fibroblasts and improves fibroblast migration and attachment through its scaffold-like fibrillar structure.
4. It also creates a throbogenic surface that stimulates platelet attachment which may accelerate fibrin and clot attachment.
5. Collagen is a hemostatic, a property that enhances wound healing.

In conclusion, the finding of this study indicated that the use of GTR technique (collagen membrane) in combination with xenogenic bone graft material was beneficial for the treatment periodontal intrabony defects. This combination technique provided improved outcomes in terms of clinical, radiographic, and intrasurgical parameters. Further studies are required with larger sample size and longer follow-ups.
